# Acute Heart Failure in the Course of Macrophage Activation Syndrome Due to Newly Diagnosed Systemic Lupus Erythematosus: Case Presentation and Literature Review

**DOI:** 10.3390/medicina60030392

**Published:** 2024-02-26

**Authors:** Jakub Kuna, Grzegorz Chmielewski, Łukasz Jaśkiewicz, Magdalena Krajewska-Włodarczyk

**Affiliations:** 1 Department of Rheumatology, School of Medicine, Collegium Medicum, University of Warmia and Mazury, 10-900 Olsztyn, Poland; gchmielewski.gc@gmail.com (G.C.); lukasz.jaskiewicz@uwm.edu.pl (Ł.J.); 2 Department of Human Physiology and Pathophysiology, School of Medicine, Collegium Medicum, University of Warmia and Mazury in Olsztyn, 10-082 Olsztyn, Poland

**Keywords:** macrophages, macrophage activation syndrome, systemic lupus erythematosus, heart failure

## Abstract

Macrophage activation syndrome is an uncommon yet dangerous and potentially fatal complication of many rheumatic diseases, inducing multiple organ failure, including, although rarely, acute heart failure. In the following paper, we present a case of a 37-year-old woman who, in a short period of time after a gynecological procedure due to fetal death, developed full-blown lupus erythematosus leading to early stages of macrophage activation syndrome with acute heart failure as its main clinical manifestation. We also include herein a brief literature review of the current understanding of diverse macrophage populations and their functions in various organs (focusing especially on the heart muscle), as well as a summary of different attempts at composing concise criteria for diagnosing macrophage activation syndrome.

## 1. Introduction

Macrophage activation syndrome (MAS) is a dangerous yet difficult-to-diagnose disorder linked to numerous systemic diseases of the connective tissue—systemic lupus erythematosus (SLE), systemic juvenile idiopathic arthritis (sJIA), rheumatoid arthritis (RA), and systemic sclerosis (SSc), among others. MAS belongs to the acquired (secondary) hemophagocytic lymphohistiocytoses (sHLH) group, and its diagnosis criteria are based on the better-researched and better-described familial (primary) lymphohistiocytoses (fHLH), belonging to the hematology domain. This causes some diagnostic problems as those criteria do not always adhere to the image observed in the course of connective tissue diseases. Moreover, in diseases such as SLE or sJIA, which can present with a wide variety of symptoms, detecting a developing MAS presents additional clinical challenges, especially in differentiating MAS from other conditions often co-occurring with an aggravation of rheumatoid disorders, notably septic infections or inflammations following surgical intervention [1]. An accurate diagnosis and speedy introduction of appropriate treatment is key and can be the deciding factor between the patient’s life or death. Epidemiological data indicate the prevalence of MAS from 0.9 to 4.6% of SLE cases and about 10% in the course of sJIA (and up to 40% in subclinical MAS), and the mortality rate in sJIA patients ranges from 8 to 23%, while in SLE patients it is 5 to 35% (up to 50% in adults) [2]. A frequent cause of death in the course of MAS is complications connected with cardiac involvement.

The case described below is of a patient suffering from SLE developed after a gynecological surgery, followed by symptoms suggesting a developing MAS complicated by acute heart failure.

## 2. Case Presentation

This study describes the case of a 37-year-old woman who was initially admitted to the Gynecology and Obstetrics Ward with a mounting fever (up to 39.8 °C) accompanied by generalized arthralgia (without joint swelling), muscle fatigue, and dyspnoea after minimal physical effort—the symptoms appeared 6 days after curettage of the uterine cavity due to fetal death in the 9th HBD. Lab analysis results indicated pancytopenia with moderate microcytic anemia and the presence of mielocytes in the peripheral blood smear, an increased concentration of C-reactive protein (CRP) with procalcitonin at normal levels, D-dimers, and ferritin. Microbiological analysis yielded a positive result in the vaginal swab culture, where Acinetobacter baumanii was observed. Due to persisting dyspnoea, the patient was initially suspected of pulmonary embolism, yet the ECG did not indicate signs of right ventricle (RV) overload or left ventricle (LV) contraction dysfunction. In echocardiography (ECHO), the ejection fraction was estimated at 65%, and no fluid was detected in the pericardium. Angio-CT imaging of the chest area did not indicate the presence of embolic material; however, it did detect the presence of an 11 mm effusion in the pleural cavities. The treatment included targeted antibiotic therapy in the form of gentamycin and clindamycin. After the treatment yielded only a minimal improvement in the general condition of the patient, despite sterile blood cultures, the diagnostic procedures were deepened—a bone marrow biopsy smear indicated reactive changes and the presence of forms containing a homogenous, eosinophilic, amorphous nuclear substance (like in systemic connective tissue disorders), and further laboratory tests detected hypoalbuminemia, a decreased concentration of the C3 and C4 complement proteins, and the presence of antinuclear antibodies of a granular fluorescence pattern at the 1:1280 titer (SS-A and SS-B present). After 16 days at the Gynecology Ward, the patient was transferred to the Rheumatology Clinic.

At the time of admission, the patient reported persisting fatigue, generalized athralgia with joint stiffness lasting for 1 h, dyspnoea after minimal physical effort, and no signs of stenocardia. Physical examination indicated a persistent subfebrile state up to 37.4 °C, muscle weakness in the limbs, reduced lung sounds in the lower lung fields, tachycardia 100/min, and moderate symmetrical swelling of the feet and shins. Lab tests indicated a progressing pancytopenia with signs of severe microcytic anemia: white blood count 3.05 × 10^3^ (4–10), hemoglobin 7.3 g/dL (13–17), platelet count 117 × 10^3^ (150–370), increased erythrocyte sedimentation rate (ESR) to 27 mm/h (<15), increased concentrations of CRP to 199 mg/dL (<5) (procalcitonin at normal levels), D-dimers to 21 μg/mL (<0.5), ferritin 953 ng/mL (10–200), N-terminal prohormone of brain natriuretic peptide (NT pro-BNP) 798 pg/mL (<125) and triglycerides 268 mg/dL (40–160), a decreased concentration of fibrinogen to 142 mg/dL (180–400) and the C3 complement protein 0.67 g/l (0.75–1.4), the presence of antinuclear antibodies at the ≥1:10,000 titer with a titer of dsDNA at 1:3200, a slightly increased level of the lupus anticoagulant, and the presence of anticardiolipin antibodies in the IgM class(24.86 U/mL (<12), proteinuria (0.7 h/24 h). There was no presence of the anti-neutrophil cytoplasmic antibodies (both p-ANCA and c-ANCA), anticardiolipin antibodies in the IgG class, or anti-beta-2 glycoprotein antibodies, creatine kinase (CK) was within normal limits; the Coombs test yielded a negative result. No pathogenic bacteria were detected in blood, urine, saliva, and anal swab cultures.

Based on the course of illness to date, laboratory test results, and image examinations, the patient was diagnosed with systemic lupus erythematosus according to the 2018 ACR and EULAR classification criteria, with a score of 24 points (history of fever, pleural effusion, proteinuria, leukopenia, presence of anti-dsDNA antibodies, and low C3 and C4 complement proteins). The medication included heparin prophylaxis, antipyretics, intravenous methylprednisolone at 1 g/day over 6 days (6 g in total), followed by oral prednisone at 1 mg/kg of body weight (patient’s weight was 55 kg).

**Figure 1:**
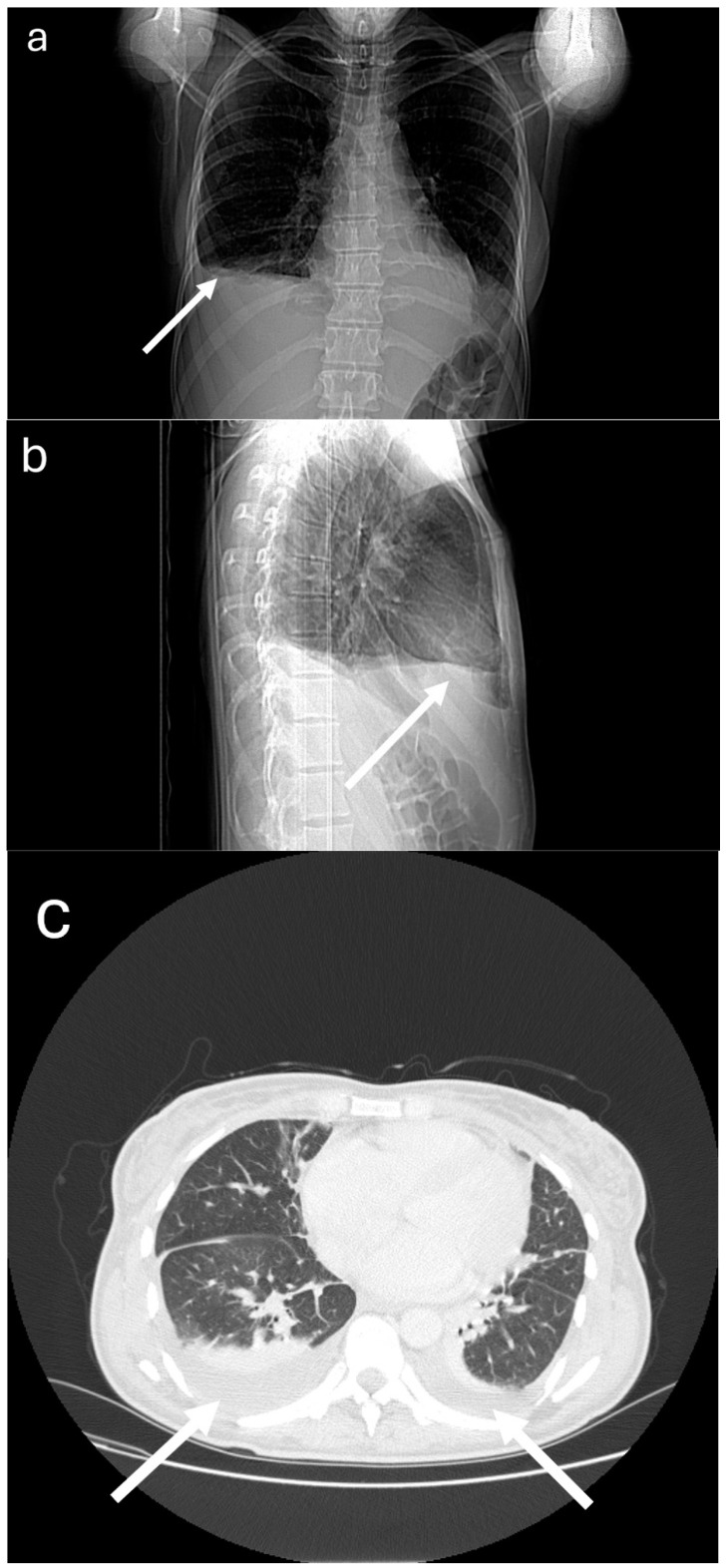
Patient’s chest CT examination: (**a**) frontal overview; (**b**) right sagittal overview; (**c**) axial scan; pleural effusion is indicated with an arrow.

Despite treatment, the patient complained about aggravated dyspnoea after minimal physical effort and pain in the right epigastrium; physical examination indicated increased swelling in the lower limbs; auscultation above the lung fields indicated reduced lung sounds in the lower fields of both lungs; tenderness in the right epigastrium but with no peritoneal symptoms or peristaltic disorders. The above was accompanied by an increasing fever. Control examinations indicated further increases in the ferritin level and decrease in fibrinogen to 94 mg/dl, worsening morphotic parameters of blood with an increasing concentration of NT pro-BNP (up to 4600 pg/mL) and troponin T (albeit within the upper limits of the norm). CT examinations detected an effusion in the pleural cavities (up to 25 mm width, as compared to 11 mm in the previous test) (Figure 1), as well as a small amount of fluid in the recto-uterine pouch; there were no irregularities in the organs of the abdominal cavity. The ECHO examination did not indicate signs of RV overload or LV contraction dysfunction; however, a small mitral valve regurgitation was detected. Using the H Score calculator to assess the risk of MAS occurring [3], the probability was estimated at 80–88% (H score of 195). In light of the above, on the fifth day of treatment, it was decided to add cyclosporin at 2 × 100 mg and an ACE inhibitor with a loop diuretic to the steroid. Initially, an increase in the body temperature to 39.1 °C was observed, along with an increased concentration of CRP and D-dimers. Repeated cultures of blood and urine were normal; however, a throat swab culture indicated the presence of Hemophilus influenzae. The treatment included intravenous antibiotics initiated in the 10th day of hospitalization (ciprofloxacin—administered for 10 days; metronidazole—for 7 days) and low-molecular-weight heparin at a weight-based dosage, as well as a transfusion of 2 units of leukocyte depleted irradiated red blood cell concentrate. After three days of cyclosporin treatment, a constant gradual decrease in ferritin concentration was observed; subsequent days showed a decrease in the NT pro-BNP concentration, and by the ninth day, the concentration of fibrinogen was within the norm. Due to adverse reactions to cyclosporin (nausea, vomiting, and loose stools), the drug was substituted after eight days of treatment by mycophenolate mofetil at 2 × 500 mg and chloroquine at 1 × 250 mg.

Over the course of the remaining hospitalization, the patient (who remained at the Rheumatology Clinic for a total of 24 days) showed a gradual increase in exertion tolerance, regression of leg swelling, complete abatement of the fever, improvement in morphotic parameters of blood in all cell cultures (only benign normocytic anemia on the day of discharge from the Clinic), normalized concentrations of NT pro-BNP and triglycerides, a reduction in CRP, ferritin and D-dimers, and a reduction in proteinuria to 0.4 g/24 h. Follow-up echocardiograms showed no abnormalities. Extended medication included mycophenolate mofetil and chloroquine; a gradual reduction in prednisone dosage was recommended (under the supervision of a rheumatologist). Due to persisting joint pain despite the above treatment, methotrexate was added in subcutaneous doses of 25 mg/week. A summary of selected clinical parameters and medications used during hospitalization was presented in Table 1.

## 3. Discussion

Macrophage activation syndrome in the course of systemic connective tissue disorders should always be taken into consideration whenever there is no marked improvement in the patient’s health after standard treatment, especially in the case of additional symptoms suggesting a potential organ failure. The frequent co-occurrence of MAS and SLE or sJIA is not coincidental. It is assumed that a triad of factors is needed to develop a full-blown cytokine storm: a genetic defect impairing the function of NK cells or CD8 cytotoxic T cells (most often a mutation of the PRF-1 gene, which encodes perforin), chronic inflammation (as in SLE and sJIA), and a triggering factor (most often an infection, sometimes a major exacerbation of the disease or treatment with various drugs, such as sulphasalazine, methotrexate, NSAIDs, adalimumab, and tocilizumab, among others) [1,4]. Cardiac involvement is not a rare occurrence in the course of this syndrome [5]; according to one of the studies on MAS in patients with sJIA, cardiac involvement was present in 25% of cases (most often in the form of pericarditis) [6], and in adult patients with MAS in the course of SLE, myocarditis was diagnosed in 21.4% of cases and pericarditis in 23.3% [7]. Yet there are not many case reports of heart failure caused by MAS available in the literature. In recent years, only a handful of such cases were reported: one paper describing a decompensated right heart failure [8], one presenting two cases of acute heart failure treated with immunosuppression [9], and one case report of fatal acute heart failure reported by our team [10].

The long-standing model of dividing macrophages into M1 (pro-inflammatory) and M2 (anti-inflammatory), albeit useful in the general, simplified understanding of their roles, turned out to be only a representation of the radical functions of these cells. Tests conducted mainly on mice (although more test results on humans are becoming available) allowed researchers to determine the structure of macrophages and discern their different populations in specific organs [11].

A study conducted in 2022 on mouse organs delineated three distinct subsets of macrophages, which differed not only in their genetic expression but also in their spatial localization within these organs. The groups were identified via combinatorial expression of four markers: the phosphatidylserine receptor T cell immunoglobulin and mucin domain containing 4 (TIMD4), lymphatic vessel endothelial hyaluronian receptor 1 (LYVE1), folate receptor beta (FOLR2), and chemokine receptor C-C motif chemokine receptor 2 (CCR2). The relation of expressions of the first three (termed TLF) to the CCR2 helped identify those macrophage populations; the first macrophage subset was called TLF+, the second CCR2+, and finally, the third subset of macrophages was termed MHC-II^hi^. TLF+ macrophages play an important role in organ development and homeostasis, and this population is almost exclusively self-renewing. The CCR2+ population is continually replaced by circulating monocytes. The MHC-II^hi^ population appears to have a predetermined maximum in each organ, and its population is replenished by monocytes up to that limit [11].

Drawing from earlier studies, but specifically focusing on the heart, two main groups of macrophages were distinguished depending on their expression of the CCR receptors. Macrophages without these receptors (CCR2−) are present in the heart as early as in the fetal period, and the survival of their population is completely dependent on tissue reserves. They exhibit an increased expression of growth factors and are equipped with genes responsible for myogenesis and DNA repair, similar to the TLF+ cells mentioned above. It has been proven that they facilitate angiogenesis and inhibit fibrosis, and their depletion results in accelerated heart failure development [12]. In addition, they work to preserve cardiac output by promoting enlargement of the left ventricle and facilitating coronary angiogenesis [13]. The CCR2+ cells appear in the heart in the later stages of the fetal period; their role involves pro-inflammatory activation—the pro-inflammatory cytokines and chemokines they release participate in the recruitment of circulating monocytes and neutrophils, which strengthens the inflammatory response and replenishes the CCR2+ macrophage population in the heart after the danger ends. Extended inflammations can partially or even completely deplete this group of macrophages. Their replacement with CCR2+ cells may result in a worsening of the ongoing process, as well as subsequent inflammatory episodes and, later, an abnormal reconstruction of the myocardium. Effective treatment can interrupt this process and prevent dangerous complications [5].

Causative therapy of acute heart failure differs depending on the etiology—in differential diagnostics, one should consider not only cardiac involvement in the course of MAS but also acute coronary syndrome, pulmonary embolism, pericarditis, infectious agents, or acute valve disorders. In the case of the patient described in our study, the coronary etiology could be excluded from the start—she did not experience stenocardial pains, several measures of T troponin concentration were all within the norm, ECG did not indicate signs of ischemia, and heart echogram did not suggest any contraction dysfunctions. Pulmonary embolism could also be excluded despite a heightened level of D-dimers, based on angio-CT imaging of the chest and no signs of RV overload in the echogram. In addition, no effusions in the pericardial sac or significant changes in the morphology and function of the valves were observed. Thus, it can be surmised with a large amount of certainty that the primary cause of such a fast development of heart failure was a rapidly developing MAS, which the medication applied managed to stop. It is also possible that severe anemia was a factor intensifying the symptoms, especially given that a transfusion of red blood cell concentrate coincided with the modification of immunosuppressive treatment.

There is no doubt that a quick diagnosis and appropriate medication are key to preserving the patient’s health and life. Over the years, there have been attempts at analyzing various parameters whose deviations are significant to the course of MAS [14]. For example, the Delphi International Survey of 2011 takes into account 28 different parameters [15]. With regard to the adult population, there are no clear classification criteria for MAS. Clinical practice utilizes four main systems of classification, as follows: the criteria for fHLH established by the International Histiocyte Society in 2004 [16]; Ravelli’s criteria of 2005, which are more precise in patients with sJIA [17]; the MAS Study Group criteria of 2011 [15]; and the H Score [3] used to estimate the likelihood of MAS occurrence. Table 2 presents a summary of all these criteria (Table 2). It is worth noting the recurring laboratory parameters in these criteria, which are characteristic of developing MAS: hyperferritinemia, hypertriglyceridemia, hypofibrinogenemia, and, in one case, a decrease in ESR. Thus, it is worth remembering to check for these parameters in a patient with systemic connective tissue disease whose condition worsens despite treatment.

In the case of our patient, the H score calculator devised in 2014 was a helpful tool to determine the risk of MAS. The algorithm calculating the probability of MAS uses nine easy-to-measure parameters: patient history concerning immunosuppression, body temperature, enlarged liver or spleen, cytopenia in blood morphology tests, the concentration of ferritin, triglycerides, fibrinogen, and aspartate transaminase, as well as signs of hemophagocytes in the bone marrow. For our patient, the probability was estimated at 80–88%, which resulted in the intensification of treatment, leading to the patient’s condition improving.

There are no clear therapeutic protocols for MAS, but numerous studies have shown the effectiveness of high-dose corticosteroids, calcineurin inhibitors (cyclosporine), followed by etoposide, cyclosporine, i.v. immunoglobulins, antithymocyte globulin, or biologic drugs such as IL-1 or IL-6 inhibitors [2,8,9]. Some authors distinguish an entity similar to MAS called the macrophage activation-like syndrome (MALS), specifically in critically ill patients with sepsis rapidly progressing to an early death. The distinction is based mainly on the lack of data from bone marrow biopsy, which is often difficult to perform on those patients. MALS was proposed as an independent risk factor for 10-day mortality [18]. The proposed classification criteria for MALS were sepsis with either an H Score above 151 points or the co-occurrence of hepatobiliary dysfunction with disseminated intravascular coagulation [19].

It is worth mentioning the use of the H-score in SARS-CoV-2 patients developing HLH-like symptoms. It may be a useful tool for screening, especially in patients with hepato- and splenomegaly, progressive cytopenia, and subacute fever occurring alongside SARS-CoV-2 infection [20]. Moreover, there is a growing number of case reports of patients developing MAS even several months after COVID-19 infection, which suggests that looking for this aetiologic factor in patients’ recent history may explain the occurrence of MAS in individuals with no autoimmune comorbidities [21,22].

## 4. Conclusions

Systemic lupus erythematosus is a disease that poses significant diagnostic challenges, which may delay the application of appropriate treatment. This translates to a risk of early, potentially fatal complications, especially in the case of a particularly intense initial stage. One should always consider the risk of MAS occurrence in these cases—the multitude of classification criteria indirectly points to the difficulties in identifying this dangerous complication. In the case of our patient, using the H Score method facilitated quick therapeutic decisions, which led to the withdrawal of symptoms of acute heart failure in the course of developing MAS.

Even though none of the classification criteria for hemophagocytic lymphohistiocytoses include cardiac symptoms, it needs to be kept in mind that a developing MAS can be the cause of their occurrence.

## Figures and Tables

**Table 1:** Selected clinical parameters and medications used during hospitalization (for drugs, total daily doses in milligrams are given). Abbreviations: WBC—white blood count; HGB—hemoglobin; HCT—hematocryte; PLT—platelet count; CRP—C-reactive protein; ESR—erythrocyte sedimentation rate; NT pro-BNP—N-terminal prohormone of brain natriuretic peptide. Normal range is given in square brackets.

Hospitalization Day	1	2	5	6	7	8	12	13	14	15	16	22	23
WBC (10^3^/μL) (4–10)	3.05	2.79	5.68	6.27	4.88	6.38	3.86				3.45	6.43	7.5
HGB (g/dL) (13–17)	7.3	6.8	6.5	6.4	6.3	6.4	8.3				8.4	11.2	10.4
HCT (%) (40–52)	21.9	20.3	20.1	19.8	19.5	19.6	24.9				23.5	34	31.1
PLT (10^3^/μL) (150–370)	117	116	135	110	111	119	215				257	502	492
CRP (mg/dL) (<5)	199	169	47	40	39	16	62		80		72	38	28
ESR (mm/h) (<15)	27			29								35	
FERRITIN (ng/mL) (10–200)	953		>2000	3322	1614		654		605			623	
D-DIMER (μg/mL) (0–0.5)	21		1.8			6	12		7				5
FIBRINOGEN (mg/dL) (180–400)	142	132	94		101		271		323			396	
NT pro-BNP (pg/mL) (<125)	798				4158	4601	1836		1089			175	
TRIGLYCERIDES (mg/dL) (40–160)	268								141				
TEMPERATURE (°C)	37.4	38.7	36.9	37	36.9	37.1	38	38.7	38.7	37.2	37.2	36.8	36.7
Methyloprednisolone (i.v.)		1000	1000	1000	1000								
Prednisone (p.o.)						30	55	55	55	55	55	55	55
Cyclosporin			200	200	200	200	200						
Mycofenolate mofetil								1000	1000	1000	1000	1000	1000
Chloroquine								250	250	250	250	250	250
Methotrexate (weekly)											25		25

**Table 2:** Different classification criteria for MAS diagnosis.

fHLH Criteria [16]	Ravelli Criteria [17]	MAS Study Group Criteria [15]	H Score for Reactive Hemophagocytic Syndrome [3]
*Clinical features:* - Fever - Splenomegaly *Laboratory features:* - Cytopenias (≥2 of 3 lineages in the peripheral blood) - Hypertriglyceridemia and/or hypofibrinogenemia - Hemophagocytosis in bone marrow, spleen, or lymph nodes - Ferritin - Low or absent NK cell activity - Increased serum sIL-2Rα (at least 5 of the 8) Or A molecular diagnosis consistent with HLH (i.e., reported mutations in genes encoding either PRF1 or MUNC13-4, STX11, STXBP2, Rab27a, SH2D1A or BIRC4)	*Clinical features:* - Central nervous systemic dysfunction - Hemorrhages - Hepatomegaly *Laboratory features:* - Decreased platelet count - Elevated aspartate aminotransferase - Decreased white blood count - Hypofibrinogenemia Two or more laboratory criteria or any two or more clinical and/or laboratory criteria	*Clinical features:* - Persistent, continuous fever ≥38 °C *Laboratory features:* - Platelets - Hyperferritinemia - Liver enzymes - Leukocyte count - Falling ESR - Hypofibrinogenemia - Hypertriglyceridemia - Evidence of macrophage hemophagocytosis in the bone marrow	*Clinical features:* - Known underlying immunosuppression - Temperature - Organomegaly (liver or spleen) *Laboratory features:* - No. of cytopenias - Ferritin- Triglycerides - Fibrinogen - Aspartate aminotransferase - Hemophagocytosis features on bone marrow aspirate

## Data Availability

The analyzed data sets generated during the study are available from the corresponding author upon reasonable request.
